# CSF-Net: Cross-Stage Fusion Network with Dual Backbone for Small Object Detection

**DOI:** 10.3390/s26041387

**Published:** 2026-02-23

**Authors:** Beilei Wang, Hongyu Li, Lin Wei, Yichuan Zhang

**Affiliations:** Software College, Northeastern University, Shenyang 110819, China; wangbl@swc.neu.edu.cn (B.W.); lihongyu1@mails.neu.edu.cn (H.L.); weil2@mails.neu.edu.cn (L.W.)

**Keywords:** small object detection, cross-stage fusion, dual backbone, dynamic fusion

## Abstract

Small object detection remains a challenge in computer vision due to low pixel occupancy, feature scarcity, and susceptibility to background interference. Conventional single-backbone networks often struggle to balance deep semantic extraction with the preservation of shallow details. Deep down-sampling can lead to the loss of edge and texture information, while late-stage fusion may fail to recover these details effectively. To address these limitations, this paper proposes a Cross-Stage Fusion Network with a Dual Backbone (CSF-Net). Our network employs an asymmetric design: a shallow backbone maintains a higher resolution to preserve fine-grained details, while a deep backbone extracts contextual semantics. These two streams interact via progressive cross-stage connections, facilitating the early fusion of small object information. Experiments on the Micro RGB UAV dataset indicate that CSF-Net improves the mAP of the YOLOV8 baseline from 62.8% to 67.0%, validating the effectiveness of the proposed architecture in enhancing detection performance for small targets.

## 1. Introduction

In recent years, while general-purpose object detection has progressed significantly, small object detection remains a critical and persistent challenge in computer vision. However, within this domain, there exists an even more distinct and difficult challenge: the detection of “Micro” objects. Unlike standard small objects (e.g., those defined as <32 × 32 pixels in datasets like COCO or VisDrone), micro objects suffer from extreme information sparsity, often occupying fewer than 16×16 pixels or appearing as sub-pixel targets in high-altitude surveillance imagery. This creates unique difficulties beyond standard small object detection, including severely limited resolution, sparse contextual information, and high susceptibility to background interference [1].

The central dilemma stems from the inherent information sparsity of these micro targets. First, targets occupy minimal pixels; thus, repeated down-sampling in deep neural networks rapidly degrades or permanently destroys their critical features. Second, their diminutive scale affords little intrinsic visual distinction, distinct from the relatively richer features found in larger “small” objects typical of general benchmarks. Consequently, methods optimized for general small object datasets often fail in these extreme micro-scale scenarios due to the overwhelming dominance of background noise. In high-precision, safety-critical applications—such as autonomous driving (distant pedestrians and traffic signs) [2], remote sensing/UAV imagery (dense or distant targets), and medical image analysis (micro-lesions)—the risks of missed detections and false positives escalate sharply as target size decreases to the micro scale. This severely compromises the reliability and performance of detection systems in real-world deployments, necessitating validation on datasets specifically designed for these extreme conditions, such as the Micro RGB UAV dataset used in this study.

Small object detection faces a fundamental dilemma: targets occupy minimal pixels and lack intrinsic visual distinction. Existing research paths addressing this information sparsity all suffer from limitations. Multi-scale fusion paradigms, such as FPN [3], employ deep down-sampling strategies that often lead to the irreversible loss of critical details early in the network. Furthermore, Attention Mechanisms like CBAM [4], while mitigating interference, are constrained by static weights that lack input adaptability, causing weak signals to be submerged by background noise. Although high-resolution architectures represented by HRNet [5] preserve details, they incur prohibitive computational overhead unsuitable for edge scenarios. Consequently, current methods either lose critical details prematurely or fail to effectively suppress noise during fusion.

To address these limitations, we propose the Cross-Stage Fusion dual-backbone Network (CSF-Net). First, we design the Channel Switch (CSwitch) module to establish early cross-stage connections, transmitting shallow, high-resolution features to deep layers to prevent irreversible information loss. Second, we introduce the Dynamic Fusion (DFuse) module. Unlike static approaches, DFuse employs an input-adaptive channel screening mechanism to dynamically adjust fusion weights, ensuring valid small object signals are not overwhelmed by background noise. Finally, in the P3 detection head, we introduce the CReToNeXt module to replace the original C2f component. By employing a cross-depth dense connection structure, CReToNeXt enables fine-grained extraction and reuse of shallow details without incurring additional computational overhead. The main contributions of this paper are summarized as follows:

- We propose CSF-Net, an architecture optimized for small object detection. Our asymmetric dual-backbone strategy seeks to balance high-resolution detail preservation with structural efficiency.
- We introduce an early fusion mechanism. By coordinating early feature injection (CSwitch) with structural adaptive alignment (DFuse), we address the limitations of static fusion strategies in handling heterogeneous features.
- We refine the detection head by incorporating the CReToNeXt module, which employs a cross-depth dense connection structure to improve feature utilization.

## 2. Related Works

The fundamental challenge of small object detection lies in its intrinsic information sparsity. The minimal pixel proportion of small objects results in their feature representations being inherently weak within deep neural networks. Crucially, as this weak signal undergoes multiple irreversible down-sampling operations during forward propagation, it becomes highly susceptible to permanent loss. Furthermore, it is easily suppressed and obscured by complex background noise. To tackle these dual challenges of feature loss and noise interference, current research primarily proceeds along three main avenues: multi-scale feature fusion, high-resolution feature preservation, and contextual enhancement and Attention Mechanisms.

### 2.1. Multi-Scale Feature Fusion

To address the rapid degradation and eventual loss of small object features in deep networks, Feature Pyramid Network (FPN) was introduced, quickly becoming a standard configuration in modern object detectors. FPN constructs a top-down pathway with lateral connections, enabling the fusion of strong semantic information, extracted from deep layers, with shallow, high-resolution feature maps.

Building on this foundation, subsequent research has continuously optimized these fusion pathways. For instance, PANet [6] appended a bottom-up path to FPN’s top-down structure, creating a bi-directional fusion pathway intended to shorten the information distance between shallow layers and the top-most predictions. Subsequently, the BiFPN [7], proposed in EfficientDet, designed a more efficient bi-directional structure and introduced learnable weights, allowing the network to dynamically adjust the contribution of different feature scales during fusion.

However, the core limitation of these FPN-based paradigms lies in their “deep-down-sampling-first, reverse-fusion-later” paradigm. This design dictates that the critical edge and texture details, upon which small objects depend, are permanently lost after repeated, irreversible down sampling operations in the network’s early forward pass. Consequently, regardless of how exquisitely the subsequent fusion pathways (such as bi-directional paths or weighted fusion) are designed, they cannot recover this information that was already lost in the initial stages.

To address the inherent limitation of FPN-based paradigms, wherein information is lost prior to fusion, the Cross-Stage Fusion Network (CSF-Net) proposed in this paper employs an asymmetric dual-backbone architecture. This architecture incorporates a Shallow Backbone (e.g., based on the YOLOV8 backbone) whose primary objective is to preserve high-resolution feature representations (such as edges and textures) by minimizing downsampling operations. This design aims to mitigate the irreversible information loss that occurs in conventional network forward-pass pathways, ensuring that subsequent fusion stages can leverage high-quality shallow feature sources and thus overcoming the inability of FPN paradigms to recover lost spatial details.

### 2.2. Context Awareness and Attention Mechanisms

Another category of methods recognizes the dilemma of low intrinsic feature discriminability in small objects, turning instead to leverage surrounding contextual information to aid detection. One strategy is to enlarge the receptive field—for example, by introducing Dilated Convolutions or constructing a Spatial Pyramid Pooling [8] module—to capture a broader semantic environment, thereby helping the network infer the presence of small targets.

Concurrently, Attention Mechanisms have been widely adopted to address the problem of small object signals being dominated by background noise. By introducing channel attention (e.g., SENet [9]) or spatial attention (e.g., CBAM), these modules enable the network to adaptively enhance feature channels containing targets and suppress responses from irrelevant background regions.

The limitations of this approach lie in its application timing and fusion method. First, both context and attention modules typically operate on deep feature maps that have already undergone multiple down sampling. At this stage, the effective signal of the small object is already exceptionally weak. Attempting to enhance features with such a low signal-to-noise ratio yields limited results and may even amplify the background noise. Second, existing fusion strategies, including the weighted fusion in BiFPN, have weights that are fixed after training, meaning the fusion ratios remain constant. This “input-independent” static fusion approach cannot adapt to the varying background complexity and target sparsity of each individual image, easily leading to the small object signal being submerged in background noise.

To address the aforementioned challenges, the proposed CSF-Net incorporates two key improvements. First, rather than solely applying attention to deep features, CSF-Net utilizes the designed CSwitch module to inject high-resolution features (e.g., P3/8, P4/16) from the Shallow Backbone into the Fusion Backbone at an early stage. This approach ensures that feature enhancement operations are performed on feature maps with a higher Signal-to-Noise Ratio (SNR [10]). Second, to overcome the limitations of static fusion, the designed DFuse module implements a dynamic fusion mechanism. This mechanism adaptively adjusts the fusion weights between shallow details and deep contextual information based on the input content, thereby effectively suppressing noise interference and enhancing the feature saliency of small objects in complex backgrounds.

### 2.3. High-Resolution Feature Preservation

To fundamentally solve the problem of shallow detail loss, some research has explored architectures that maintain high-resolution feature representations throughout the network’s forward pass.

The most typical representative is HRNet (High-Resolution Network). HRNet’s core idea is to maintain multiple parallel branches, from high to low resolution, throughout the entire network, and to perform repeated, dense information exchanges between these branches. This design ensures that its output high-resolution feature map consistently carries rich semantic information that has been deeply processed. This philosophy has also inspired related architectures such as DLA [9] (Deep Layer Aggregation) and variants like Lite-HRNet, which attempt to reduce computational complexity [11].

However, the symmetric parallel design of such architectures, while maximizing detail preservation, inevitably introduces extremely high computational costs and memory occupancy. This makes them difficult to adapt to resource-constrained application scenarios, such as UAVs and autonomous driving edge computing units, which are highly sensitive to power consumption and latency.

Addressing the efficiency bottlenecks of symmetric parallel architectures, CSF-Net adopts an asymmetric dual-backbone design to balance high-resolution preservation with computational efficiency. Unlike architectures such as HRNet, CSF-Net implements an asymmetric allocation of computational resources: a lightweight Shallow Backbone is dedicated to preserving fine-grained details, while the Fusion Backbone focuses on semantic extraction and cross-stage fusion. This architectural design significantly reduces computational overhead and memory footprint while retaining critical high-resolution information, making it more suitable for deployment in resource-constrained edge computing scenarios, such as on UAVs.

### 2.4. Dual-Backbone Architecture Exploration

As an alternative approach to high-resolution preservation, dual-backbone architectures have emerged as a new direction of exploration. The core idea of these methods is typically to decouple the feature extraction task: one deep “Semantic Backbone” focuses on down sampling to capture global context, while a “Detail Backbone” maintains high resolution throughout to preserve fine-grained edge and texture information.

Despite the potential of dual-backbone [12] architectures to balance accuracy and efficiency, their bottleneck often lies in the latency of their fusion strategy. Existing dual-backbone networks mostly adopt a “Late Fusion” model. That is, the output features from the two backbones are merged only at the very end of the network (e.g., at the input of the detection head or FPN), after each has completed its respective feature extraction. This design overlooks the necessity of information interaction between the deep and shallow layers during the early and middle stages, causing the two backbones to operate almost independently and thus limiting the depth and effectiveness of the feature fusion [13].

Summary of Approach Differences. Unlike FPN-based methods that rely on late-stage fusion where details are often lost, or HRNet-based architectures that incur high computational costs, CSF-Net adopts a balanced strategy. Specifically, CSF-Net employs early cross-stage fusion (in contrast to YOLOV8’s late fusion) and maintains an asymmetric dual stream for efficiency. Furthermore, it utilizes dynamic weight generation to address the specific noise challenges of micro-UAV detection, offering a distinct advantage over static fusion methods like BiFPN.

## 3. Method

**Figure 1:**
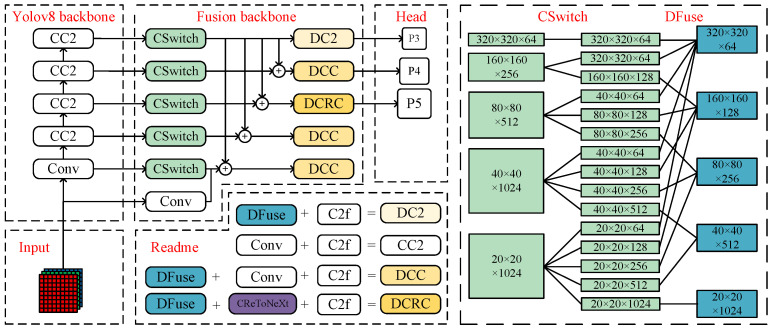
The architecture of CSF-Net. This figure illustrates the parallel structure of the YOLOV8 backbone and our designed Fusion Backbone. White modules represent native YOLOV8 components [14], while the colored modules (CSwitch, DFuse, etc.) are the novel modules proposed in this paper. The legend (“Readme” in source) in the bottom right details the internal composition of the modules in the diagram (e.g., DC2, DCC, DCRC).

To synergistically resolve the two core challenges in small object detection—the susceptibility to critical detail loss and submersion by background noise—this paper proposes a Cross-Stage Fusion dual-backbone Network (CSF-Net). As illustrated in Figure 1, CSF-Net adopts an asymmetric architecture comprising a parallel Shallow Backbone and a deep Fusion Backbone.

To ensure efficient collaboration within this asymmetric structure, we design three synergistic modules that function as a cohesive pipeline:

- **Transmission (CSwitch):** Acting as the bridge, the CSwitch module establishes early cross-stage connections. It transmits high-resolution, shallow features to the deep backbone, preventing the irreversible loss of edge and texture information typically caused by down-sampling.
- **Filtering (DFuse):** Acting as the dynamic gatekeeper, the DFuse module employs an input-adaptive mechanism. It weighs the fusion of heterogeneous features, ensuring that the faint signals of small objects transmitted by CSwitch are not overwhelmed by background noise.
- **Utilization (CReToNeXt):** Acting as the high-efficiency processor in the detection head, the CReToNeXt module utilizes cross-depth dense connections to maximize the usage of the preserved details for final bounding box regression and classification.

Shallow Backbone: We adopt the YOLOV8 backbone as the Shallow Backbone. Its core task is to maintain feature maps at a relatively high resolution (or with less down sampling) throughout its process. It focuses on forward propagation to maximally preserve the critical edge and texture details of small objects, ensuring that high-quality detail information is not diluted or permanently lost by premature down-sampling before being fed into the fusion modules.

Fusion Backbone: The deep backbone, or Fusion Backbone, concentrates on contextual semantic extraction and cross-stage fusion. It is constructed from two core modules we designed: CSwitch and DFuse.

Cross-Stage Information Interaction: Unlike traditional dual-backbone networks that only perform “Late Fusion,” the two backbones in CSF-Net engage in tight, early information interaction across multiple stages. As shown in Figure 1, feature maps output from different stages of the YOLOV8 backbone (e.g., P3/8, P4/16) are routed prematurely to the Fusion Backbone via the CSwitch module. CSwitch serves as a cross-stage connection unit, reorganizing the channels of the high-resolution features from the shallow backbone, allowing them to be efficiently injected into the corresponding levels of the deep backbone. Subsequently, the DFuse module, at each stage of the deep backbone, is responsible for receiving and dynamically fusing the detail features from the shallow backbone (via CSwitch) with the deep backbone’s own contextual features, thereby achieving fine-grained feature enhancement.

**Module Annotations:** Key components are distinctly color-coded for clarity: CSwitch (green) manages cross-stage channel distribution, while DFuse (blue) executes scale-adaptive fusion.

**Simplified Legends:** The internal structures of complex blocks (e.g., the DCRC head) are detailed in the “Legend” panel to avoid graphical saturation in the main data flow.

**Workflow:** The arrows clearly depict the layer-wise interaction where shallow details are injected into the deep backbone via CSwitch and aligned by DFuse, culminating in the P3-P5 detection heads.

Figure 1 details the asymmetric architecture of CSF-Net. It primarily consists of the Shallow Backbone (YOLOV8, composed of “Conv” and “CC2” modules) and the Deep Backbone (Fusion backbone) processing inputs in parallel. The Deep Backbone receives features from the corresponding levels of the Shallow Backbone via the “CSwitch” module. The “⊕” symbol represents element-wise addition, which is the core fusion operation. The network’s “Head” generates detection results at three different scales (P3, P4, P5). The legend (Redeme) area defines the specific composition of compound modules; for example, “DCRC” is defined as a concatenation of “DFuse + CReToNeXt + C2f”. The diagram in the bottom right visually explains the channel-splitting function of “CSwitch” and the multi-scale alignment fusion mechanism of “DFuse”.

### 3.1. CSwitch

To allocate channel quotas to features of different scales on-demand, the CSwitch module first processes the output feature map from the backbone using a core linear convolutional unit. Let the input feature map at scale *s* be

$$h_{s}\in R^{N\times C_{\mathrm{in}}\times H_{s}\times W_{s}}$$

where *N* denotes the batch size, $C_{\mathrm{in}}$ is the number of input channels, and $H_{s},W_{s}$ represent the spatial dimensions. The module first performs a 1×1 convolution to expand the number of input channels from $C_{\mathrm{in}}$ to a total of $C_{\Sigma }$ in an aggregated feature space:

(1) $$z_{s}={\mathrm{Conv}}_{1\times 1}\left(h_{s}\right)\in R^{N\times C_{\Sigma }\times H_{s}\times W_{s}}$$

The predefined channel partition list (e.g., [64,128,256]) is empirically set to align with the channel widths of the corresponding stages in the YOLOV8-nano backbone. This configuration ensures that the feature volume injected from the shallow backbone matches the capacity of the deep fusion backbone at each stage. While these values are optimized for the current model scale, the partition strategy is modular and can be scaled proportionally for larger backbones.

Subsequently, the aggregated feature map $z_{s}$ is precisely split along the channel dimension into *m* branches:

(2) $$z_{1},z_{2},\ldots ,z_{m}=\mathrm{Split}\left(z_{s},c_{1},c_{2},\ldots ,c_{m},\dim=1\right)$$

This operation splits $z_{s}$ along the channel dimension (dim = 1) according to the partition list $c_{t}$, where the respective branches have $c_{1},c_{2},\ldots ,c_{m}$ channels.

For example, for an input with $C_{\mathrm{in}}=320$ and a predefined partition list $c_{t}=\left[64,128,256\right]$, CSwitch first expands the channels to a total of 448, then splits them into three branches with 64, 128, and 256 channels, respectively.

This aggregate-and-split strategy holds significant advantages over the standard method of applying independent 1×1 convolutions per branch. First, the CSwitch module substantially enhances computational efficiency. By replacing *m* separate operations with a single shared 1×1 convolution, it drastically reduces both parameter count and computational complexity (GFLOPs). Second, this design enables Shared Representation Learning; the shared 1×1 kernel functions as a unified transformer, learning a more expressive and generalizable common representation prior to feature distribution. This shared representation benefits all *m* branches, improving final model performance while precluding the need to learn *m* independent and potentially redundant feature sets.

### 3.2. DFuse

We propose the DFuse module to achieve robust multi-scale feature fusion within the Fusion Backbone. We clarify that the “Dynamic” nature of DFuse refers to its **structural adaptability**—specifically, the capacity to accept an arbitrary number of input branches with varying resolutions and channel depths and dynamically align them to a target reference scale—rather than the generation of input-conditioned weight kernels.

Let the input feature maps be a list $X=\{X_{1},X_{2},\ldots ,X_{M}\}$, where each $X_{i}\in R^{C_{i}\times H_{i}\times W_{i}}$. The last element $X_{M}$ serves as the *reference feature* ($X_{ref}$), which determines the target channel count $C_{tgt}$ and spatial resolution $\left(H_{tgt},W_{tgt}\right)$.

The fusion process involves two distinct stages: *Channel Projection* and *Spatial Alignment*. First, for each input feature $X_{i}$ (where i<M), if its channel dimension $C_{i}$ differs from $C_{tgt}$, a learnable 1×1 convolution layer is applied to project it into the target feature space. This operation acts as a semantic alignment projection:

(3) $${\tilde{X}}_{i}^{channel}=\left\{\begin{array}{cc} {\mathrm{Conv}}_{1\times 1}^{C_{i}\to C_{tgt}}\left(X_{i}\right), & \mathrm{if}\;C_{i}\neq C_{tgt}\\ X_{i}, & \mathrm{if}\;C_{i}=C_{tgt} \end{array}\right.$$

Second, to handle resolution mismatch, nearest-neighbor interpolation is employed to align the spatial dimensions of all branches to $\left(H_{tgt},W_{tgt}\right)$:

(4) $${\tilde{X}}_{i}=\mathrm{Interpolate}\left({\tilde{X}}_{i}^{channel},\mathrm{size}=\left(H_{tgt},W_{tgt}\right)\right)$$

Finally, the aligned features are fused via element-wise summation to produce the output *Y*:

(5) $$Y=\sum_{i=1}^{M}{\tilde{X}}_{i}$$

This concise design allows CSF-Net to efficiently aggregate high-resolution details from the shallow backbone with semantic contexts from the deep backbone without the excessive computational overhead associated with dynamic weight generation networks. The detailed procedure is outlined below.

### 3.3. CReToNeXt

To enhance the feature representation capability of the P3/8 scale detection head for small objects, we introduce the CReToNeXt module to replace the original C2f component. This module aims to achieve superior fusion of details and semantics through a cross-depth dense connection structure, all while incurring minimal computational overhead.

The CReToNeXt module first maps the input feature map $X\in R^{N\times C_{\mathrm{in}}\times H\times W}$:

(6) $$X_{s}={\mathrm{Conv}}_{1\times 1}\left(X\right)\in R^{N\times C_{\mathrm{first}}\times H\times W}$$

(7) $$X_{d_{0}}={\mathrm{Conv}}_{1\times 1}^{d}\left(X\right)\in R^{N\times C_{\mathrm{mid}}\times H\times W}$$

where $C_{\mathrm{first}}$ and $C_{\mathrm{mid}}$ are the channel counts of the shallow and deep branches, respectively.

Subsequently, the initial feature of the deep branch, $X_{d_{0}}$, is fed into a sequence composed of *n* cascaded residual blocks ($B_{i}$). The key to this module lies in its “cross-depth” connection mechanism: the output of every residual block in the sequence, $X_{d_{i}}$, is preserved:

(8) $$X_{d_{i}}=B_{i}\left(X_{d_{i-1}}\right)\;\mathrm{for}\;i=1,\ldots ,n$$

After the deep branch processing is completed, the module concatenates the shallow shortcut feature $X_{s}$ with all intermediate outputs from the deep branch, $\left\{X_{d_{1}},\ldots ,X_{d_{n}}\right\}$, along the channel dimension:

(9) $$X_{\mathrm{cat}}=\mathrm{Concat}\left(X_{s},X_{d_{1}},\ldots ,X_{d_{n}}\right)$$

(10) $$X_{\mathrm{cat}}\in R^{N\times (C_{\mathrm{first}}+n\times C_{\mathrm{mid}})\times H\times W}$$

Finally, a 1 × 1 convolution, ${\mathrm{Conv}}_{1\times 1}$, fuses this rich set of features and adjusts the channel dimension back to the target output $C_{\mathrm{out}}$:

(11) $$Y={\mathrm{Conv}}_{1\times 1}\left(X_{\mathrm{out}}\right)\in R^{N\times C_{\mathrm{out}}\times H\times W}$$

Distinct from standard CSP or C2f blocks which rely on a split-transform-merge strategy, CReToNeXt introduces a dense cross-depth shortcut. By explicitly concatenating the pristine shallow features ($X_{s}$) with the output of every subsequent transformation block, our module enforces a stronger feature reuse mechanism. This ensures that the faintest signals of small objects, often washed out in deep sequential processing, are preserved and recombined, offering a more robust representation than standard residual structures.

## 4. Results

### 4.1. Datasets and Experimental Setup

**Figure 2:**
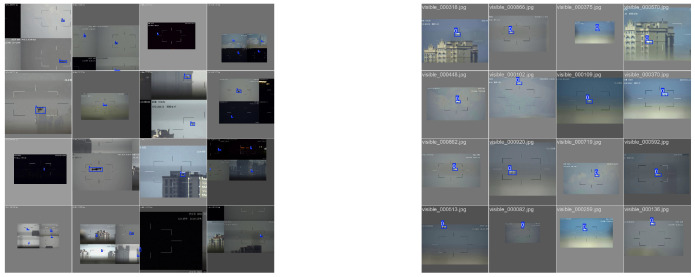
Micro RGB UAV dataset.

**Dataset.** Our experiments were conducted on the Micro RGB UAV dataset. This dataset comprises 182 training videos and 62 validation videos; notably, each video contains 50 frames (Figure 2).

This dataset was constructed specifically to address the unique challenges of aerial micro-UAV detection and tracking. As shown, the data encompasses a variety of complex and realistic observation scenarios, including bright skies with high dynamic range, cluttered urban structures, and low-light nighttime environments. The core challenge of this dataset lies in the extremely small pixel footprint of the targets, diverse backgrounds, and the presence of rapid motion and viewpoint changes, aiming to rigorously evaluate algorithm robustness and accuracy in real-world UAV applications.

**Backbone and Benchmarking.** To validate CSF-Net’s effectiveness, we selected the YOLO architecture for evaluation. The mature YOLOV8 model served as the primary baseline on the Micro RGB UAV dataset, against which we measured CSF-Net’s performance gains. **Implementation details.** All experiments were conducted using the YOLOV8 framework. The model was initialized with yolov8n.pt, an input resolution of 640 × 640, and a batch size of 32. We employed the AdamW optimizer with a cosine annealing learning rate scheduler. Models were trained for 80 epochs, including a 3-epoch warm-up. Mixed precision and early stopping were enabled. Data augmentations included HSV color perturbation, lightweight geometric perturbations, mosaic, mixup, and copy-paste. Mosaic was disabled for the final 15 epochs; all other settings followed the YOLOV8 defaults.

### 4.2. Model Correctness and Reliability Experiment

To comprehensively assess the correctness and reliability of the proposed CSF-Net architecture, we conducted a series of rigorous internal analyses, focusing on its intrinsic learning mechanism and structural design. These experiments deliberately excluded performance comparisons with external models. Instead, we focused solely on verifying the stability of the training process, the effectiveness of the internal components, and the network’s expected response to varying input conditions.

All experiments were conducted using YOLOv8 (version 8.0.22) based on Python 3.9.18 and PyTorch 2.1.0. The model was initialized with yolov8n.pt, an input resolution of 640 × 640, and a batch size of 32. We employed the AdamW optimizer with a cosine annealing learning rate scheduler.

#### 4.2.1. Training Convergence and Learning Dynamics Analysis

**Figure 3:**
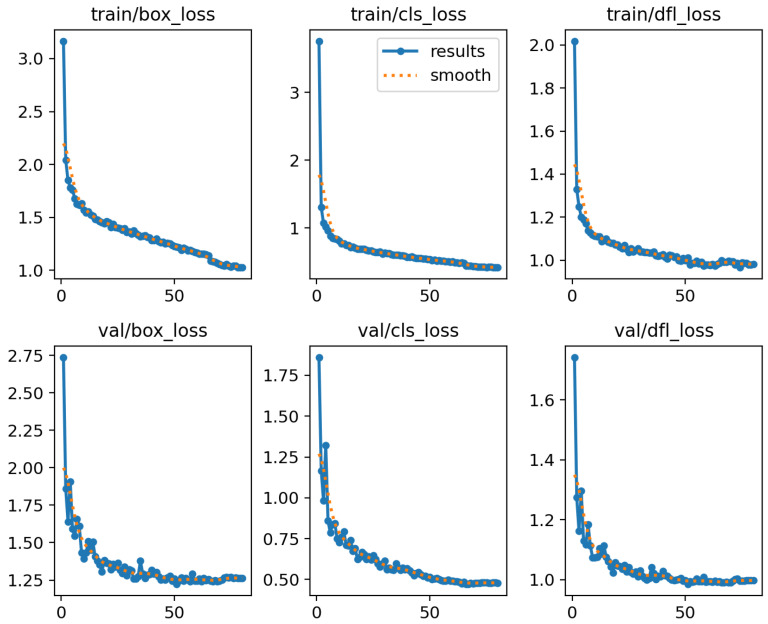
Training convergence curves of CSF-Net on the Micro RGB UAV Dataset. This figure illustrates the evolution of key loss functions for the CSF-Net model across the entire training duration. Specifically, the top row of sub-figures presents the metrics measured on the training set (train_loss), while the bottom row corresponds to the validation set metrics (val_loss).

A well-designed neural network model should exhibit a stable training process. To validate the structural stability of the CSF-Net architecture, we analyzed the training logs on the Micro RGB UAV dataset, as demonstrated by the convergence curves in Figure 3.

As shown in Figure 3, key metrics—including Bounding Box Loss (box_loss) and Classification Loss (cls_loss)—exhibit a steep decline during the initial training phase, followed by a smooth convergence. This behavior confirms that the integration of the dual-backbone components (CSwitch and DFuse) does not disrupt the network’s gradient flow or learning dynamics. The synchronized trends between training and validation losses further verify that the model maintains stable generalization capabilities without significant overfitting during the training process.

Following the initial rapid decline, all loss curves enter a prolonged, smooth convergence phase, with no significant oscillations or divergence observed throughout the process. This strongly demonstrates the high stability of the entire training procedure. Notably, even with the introduction of the new dynamic fusion module (DFuse), all loss metrics, including the dfl_loss, maintain this consistent trend of rapid convergence and smooth stabilization. This consistency validates that the integration of the DFuse module does not interfere with the network’s original learning dynamics; instead, its internal parameters are effectively learned and optimized, successfully embedding the module into the end-to-end learning pipeline.

Furthermore, by comparing the training loss and validation loss (exemplified by box_loss and cls_loss), we observe that the two curves exhibit highly synchronized trends and converge to very close final values, consistently maintaining a minimal gap. This robust observation indicates that CSF-Net possesses excellent generalization ability, effectively preventing overfitting to the training data. Collectively, these analyses fundamentally confirm the correctness and reliability of the proposed model architecture.

#### 4.2.2. Model Robustness Analysis

To evaluate the robustness of CSF-Net under adverse conditions, we introduced Gaussian noise of varying intensities to the validation set images. The results are presented in Table 1.

As shown in Table 1, CSF-Net demonstrates resilience at moderate noise levels (σ=15,30), maintaining a relatively high mAP (62.9% at σ=30). However, at high noise intensity (σ=50), the mAP drops sharply to 5.8%, while the Recall remains high at 95.2%. This divergence indicates a specific limitation of the architecture under extreme degradation: while the model retains the ability to detect the presence of objects (high recall), the severe signal-to-noise ratio compromises its ability to regress accurate bounding boxes (low precision). This suggests that the current fusion mechanism, while effective for feature retention, faces challenges in precise localization when the input signal is heavily corrupted.

### 4.3. Compared with Other Methods

To comprehensively evaluate the effectiveness of the proposed CSF-Net architecture, we conduct a rigorous performance comparison against various state-of-the-art object detection methods on the Micro RGB UAV dataset. The selected baselines include the YOLO series (e.g., YOLOV8, YOLOv7) and other advanced detectors such as TOOD, Deformable DETR, and YOLOF. All methods are evaluated under identical settings, with mean Average Precision serving as the primary performance metric. The comparative results are presented in Table 2.

As shown in Table 2, our CSF-Net (Ours) achieves 67.0% mAP, outperforming the YOLOV8 baseline (62.8%) by 4.2 percentage points. This improvement is primarily attributed to the asymmetric dual-backbone strategy, which preserves shallow details that are often lost in single-backbone down-sampling. Regarding model complexity, CSF-Net utilizes **6.87 million parameters and 18.25 GFLOPs**. Compared to the YOLOV8n baseline (4.14 million parameters and 8.06 GFLOPs), our model incurs an increase in computational cost (1.6× parameters). However, this design avoids the significantly higher overhead associated with fully dense architectures like HRNet, offering a structural compromise between detail preservation and complexity.

To accurately evaluate the overhead introduced by our dual-backbone architecture, we conducted a rigorous quantitative comparison between CSF-Net and the YOLOV8 baseline. Specifically, CSF-Net utilizes **6.87 million parameters and 18.25 GFLOPs**. Compared to the ultra-lightweight YOLOV8 baseline (4.14 million parameters and 8.06 GFLOPs), our model incurs a noticeable increase in computational cost (approximately 1.6× parameters and 2.2× GFLOPs).

For a fair and consistent comparison, all performance metrics for competing methods are sourced from reference(A video object detector with Spatio-Temporal Attention Module for micro UAV detection) [15], where these models were retrained and evaluated under identical conditions, including the dataset, hardware, and hyperparameters.

### 4.4. Ablation Studies

We conducted a series of ablation studies to systematically validate the contribution of each core component in CSF-Net. Using the full CSF-Net model (67.0% mAP) as the baseline, we individually removed or replaced key modules to assess their impact. Results are presented in Table 3.

**1. Synergy of Dual-Backbone (CSwitch + DFuse):** The exclusion of CReToNeXt isolates the dual-backbone subsystem. This configuration achieves 66.1% mAP, substantially surpassing the YOLOV8 baseline (62.8%). This substantiates that the synergy between early transmission (CSwitch) and dynamic fusion (DFuse) inherently yields robust feature representations, independent of head-level optimizations.

**2. Dependency on Transmission (DFuse + CReToNeXt):** The removal of CSwitch results in a 1.0% performance drop. This indicates that without the explicit injection of high-resolution spatial details, the downstream filtering (DFuse) and utilization (CReToNeXt) modules lack effective input. It underscores that the pipeline’s efficacy is fundamentally constrained by the quality of initial feature transmission.

**3. Necessity of Dynamic Filtering (CSwitch + CReToNeXt):** The “-DFuse” variant yields the lowest performance (65.9%) among all configurations. This suggests that direct transmission of shallow features to the detection head, bypassing intermediate refinement, introduces detrimental background noise. Consequently, DFuse is proven critical for maximizing the signal-to-noise ratio during cross-stage interaction. **CSwitch**. The CSwitch module is the core component enabling cross-stage connections, transferring high-resolution details from the shallow to the deep backbone at early stages. In this experiment, we removed all CSwitch modules, forcing the network to rely solely on late fusion. This removal caused a 1.0% mAP drop, which suggests that the absence of early interaction forces the backbones to operate independently, leading to insufficient information exchange.

**DFuse**. As shown in Table 3, this caused a 1.1 point drop in mAP. This empirical observation supports the hypothesis that dynamic fusion is more effective than static operations for processing heterogeneous features.

Thus, DFuse and CSwitch are highly complementary. CSwitch is responsible for preserving and transferring high-resolution details from the shallow stream, preventing their premature loss. However, as our ablation confirms, these raw details are inherently “noisy”—they carry abundant background noise that can easily submerge the sparse object signals. This is precisely where DFuse intervenes. It acts as the critical refinement stage for these heterogeneous features, applying its input-dependent mechanism to adaptively adjust fusion weights, suppress interference, and avoid this signal dilution. In essence, CSwitch provides the what (the high-resolution details), while DFuse provides the how (the safe, dynamic fusion). Together, they establish a complete information pathway for early preservation and dynamic refinement, allowing our asymmetric architecture to effectively utilize both detailed and semantic information, which is critical to achieving the significant mAP improvement.

**CReToNeXt**. The CReToNeXt module was introduced to replace the native C2f component in the P3/8 detection head, aiming to boost fine-grained feature extraction for tiny objects. To verify its contribution, we reverted to the original C2f module. As shown in Table 3, this caused a 0.9% mAP drop. This indicates that although C2f is efficient, CReToNeXt’s “cross-depth” dense connectivity more effectively fuses shallow details with deep semantics. For the P3 head, which specializes in small objects, CReToNeXt’s enhanced feature reuse offers a distinct advantage at a similar computational cost.

This result also reveals the crucial synergy between CReToNeXt and the overall CSF-Net pipeline. While CSwitch and DFuse are designed to preserve and transfer high-fidelity features to the P3 head, this ablation study confirms that the head’s own internal processing capability (its “utilization efficiency”) is just as vital. The experiment demonstrates that without CReToNeXt’s robust “cross-depth” dense fusion, the rich detail information meticulously preserved by the shallow backbone is ultimately under-utilized or even overwhelmed by noise at the point of detection, leading to sub-optimal performance. Thus, CSwitch and DFuse solve the delivery problem, but CReToNeXt solves the consumption problem. It is the component that ensures the high-resolution information gain from our dual-backbone design is fully maximized and translated into detection accuracy, rather than simply being present in the final feature map.

## 5. Conclusions

The proposed CSF-Net addresses the challenges of early detail loss and background interference in small object detection through its CSwitch and DFuse modules. The method achieves a 4.2% mAP improvement over the YOLOV8 baseline on the Micro RGB UAV dataset. Our ablation studies validate that coordinating early feature injection with structural alignment effectively enhances performance. Despite these gains, limitations remain, particularly regarding localization precision under extreme noise conditions (σ=50). Future work will focus on two directions: (1) exploring lightweight super-resolution modules to enhance sub-pixel target detection; and (2) applying quantization and pruning techniques to optimize the dual-backbone architecture for potential deployment on resource-constrained edge devices.

## Figures and Tables

**Table 1:** CSF-Net performance under varying Gaussian noise intensities.

σ	mAP (%)	Recall (%)
0	67.0	97.7
15	66.6	97.6
30	62.9	97.2
50	5.8	95.2

**Table 2:** Comparison with state-of-the-art methods.

Methods	mAP (%)
YOLOF	56.9
Deformable DETR	55.4
TOOD	61.3
YOLOV7	61.3
YOLOV8	62.8
QueryDet	58.1
SPPNet	49.4
RFLA	60.1
SELSA	46.7
Temporal RoI Align	42.3
TransVOD	50.5
**Ours**	**67.0**

Note: Bold font indicates the method proposed in this paper.

**Table 3:** Ablation studies of core components.

Methods	mAP(%)	Δ mAP(%)
CSF-Net (Full)	67.0	-
-CSwitch	66.0	−1.0
-DFuse	65.9	−1.1
-CReToNeXt	66.1	−0.9

## Data Availability

The source code and implementation details of CSF-Net are available on GitHub at https://github.com/lhy758/CSF-Net (accessed on 10 January 2026). The Micro RGB UAV dataset used in this study is available from the corresponding author upon reasonable request. The dataset is also accessible through the project repository at https://github.com/lhy758/CSF-Net (accessed on 10 January 2026).

## References

[1] Nikouei M., Baroutian B., Nabavi S., Taraghi F., Aghaei A., Sajedi A., Moghaddam M.E. (2025). Small Object Detection: A Comprehensive Survey on Challenges, Techniques and Real-World Applications. arXiv.

[2] Li M., Liu X., Chen S., Yang L., Du Q., Han Z., Wang J. (2024). MST-YOLO: Small object detection model for autonomous driving. Sensors.

[3] Yuan Z., Gong J., Guo B., Wang C., Liao N., Song J., Wu Q. (2024). Small object detection in uav remote sensing images based on intra-group multi-scale fusion attention and adaptive weighted feature fusion mechanism. Remote Sens..

[4] Woo S., Park J., Lee J.Y., Kweon I.S. (2018). CBAM: Convolutional block attention module. Computer Vision—ECCV 2018.

[5] Sun K., Xiao B., Liu D., Wang J. (2019). Deep high-resolution representation learning for human pose estimation. IEEE/CVF Conference on Computer Vision and Pattern Recognition.

[6] Liu S., Qi L., Qin H., Shi J., Jia J. (2018). Path aggregation network for instance segmentation. IEEE Conference on Computer Vision and Pattern Recognition.

[7] Tan M., Pang R., Le Q.V. (2020). Efficientdet: Scalable and efficient object detection. IEEE/CVF Conference on Computer Vision and Pattern Recognition.

[8] Nie L., Li B., Jiao F., Shao J., Yang T., Liu Z. (2023). ASPP-YOLOv5: A study on constructing pig facial expression recognition for heat stress. Comput. Electron. Agric..

[9] Hu J., Shen L., Sun G. (2018). Squeeze-and-excitation networks. IEEE Conference on Computer Vision and Pattern Recognition.

[10] Shannon C.E. (1948). A mathematical theory of communication. Bell Syst. Tech. J..

[11] Song Y., Yang C. (2025). DS_FusionNet: Dynamic Dual-Stream Fusion with Bidirectional Knowledge Distillation for Plant Disease Recognition. arXiv.

[12] Xu G., Wu X., Liao W., Wu X., Huang Q., Li C. (2025). DBF-Net: A Dual-Branch Network with Feature Fusion for Ultrasound Image Segmentation. 2025 IEEE International Conference on Image Processing (ICIP).

[13] Yaseen M. (2024). What is YOLOv9: An in-depth exploration of the internal features of the next-generation object detector. arXiv.

[14] Shamshoum Y., Hodos N., Sieradzki Y., Schuster A. (2025). Compact: Compressed activations for memory-efficient llm training. Proceedings of the 2025 Conference of the Nations of the Americas Chapter of the Association for Computational Linguistics: Human Language Technologies (Volume 1: Long Papers).

[15] Xu H., Ling Z., Yuan X., Wang Y. (2024). A video object detector with Spatio-Temporal Attention Module for micro UAV detection. Neurocomputing.

